# Effectiveness and cost-effectiveness of guided self-help for depression for autistic adults: the Autism Depression Trial (ADEPT-2) – protocol for a multicentre, randomised controlled trial of a remotely delivered low-intensity intervention

**DOI:** 10.1136/bmjopen-2024-084729

**Published:** 2024-11-19

**Authors:** Holly Emily Mckeon, Leonora Cotton, Rona Aldridge, Alison Cape, Madeleine Clout, Kate Cooper, Dave Dagnan, Ed Dawn, Jessica Frost, Aikaterini Georgakopoulou, Kirsty Garfield, Jeremy Horwood, Barry Ingham, Vicky Jervis, David Kessler, Peter Langdon, Chris Metcalfe, Dheeraj Rai, Alba Realpe, Christine Russell, Hannah Sheridan, Karolina Slowinska, Joanna Thorn, Liping Wen, Nicola Wiles, Ailsa Russell

**Affiliations:** 1 Bristol Trials Centre, University of Bristol, Bristol, UK; 2 Cardiff and Vale Integrated Autism Services, Cardiff and Vale University Health Board, Cardiff, UK; 3 Avon and Wiltshire Mental Health Partnership NHS Trust, Bristol, UK; 4 Department of Psychology, University of Bath, Bath, UK; 5 Community Learning Disability Team, Cumbria Northumberland Tyne and Wear NHS Foundation Trust, Newcastle upon Tyne, UK; 6 Public Contributors of ADEPT-2, Patient and Public Involvement Group, University of Bristol, Bristol, UK; 7 Department of Population Health Sciences, University of Bristol, Bristol, UK; 8 Durham Tees Valley Adult Autism Team, Tees Esk and Wear Valleys NHS Foundation Trust, Darlington, UK; 9 Social Policy and Psychology, University of Birmingham, Birmingham, UK; 10 Coventry and Warwickshire Partnership NHS Trust, Coventry, UK; 11 Centre for Academic Mental Health, School of Social & Community Medicine, Avon and Wiltshire Mental Health Partnership NHS Trust, Bristol, UK; 12 NIHR Bristol Biomedical Research Centre, University Hospitals Bristol and Weston NHS Foundation Trust, Bristol, UK

**Keywords:** Clinical Trial, Patient Reported Outcome Measures, Depression & mood disorders

## Abstract

**Introduction:**

Depression is three to four times more prevalent in autistic people and is related to reduced quality of life. There is a need for empirically supported psychological interventions for depression specifically adapted to meet the needs of autistic adults. ADEPT-2 aims to establish the clinical and cost-effectiveness of an adapted low-intensity psychological intervention (guided self-help) for depression in autistic adults.

**Methods and analysis:**

A two parallel-group multicentre pragmatic randomised controlled trial investigating the effectiveness of GSH for depression in autistic adults. Participants (n=248) aged ≥18 years with a clinical diagnosis of autism currently experiencing depression will be randomised to GSH or treatment as usual (TAU). GSH is a low-intensity psychological intervention based on the principles of behavioural activation adapted for autism. GSH comprises informational materials for nine individual sessions facilitated online by a GSH coach who has received training and supervision in delivering the intervention. The primary outcome will be Beck Depression Inventory-II depression scores at 16 weeks post randomisation with follow-up measures at 32 and 52 weeks. Additional measures of anxiety, patient-rated global improvement, quality of life, work and social adjustment, positive and negative affect will be measured 16 and 52 weeks post randomisation. The primary health economic analysis will assess the cost-effectiveness of GSH compared with TAU over 52 weeks, from a societal perspective including the National Health Service, personal social services, personal expenses, voluntary services and productivity. An embedded qualitative study will explore the acceptability, experiences and adherence of participants and therapists to treatment principles.

**Ethics and dissemination:**

This trial has been approved by the East of England - Essex Research Ethics Committee on 10 June 2022 (REC Reference number: 22/EE/0091). The findings of the research will be submitted for publication in peer-reviewed journals and disseminated in an appropriate format to trial participants and the wider public.

**Trial registration number:**

ISRCTN17547011.

**Strengths and limitations of this study**

- This pragmatic study is enabled for remote conduct and is flexibly delivered to align with participants’ preferred methods of communication.
- Autistic people were involved in the development of the intervention, study documentation and communications, including an easy-read patient information leaflet.
- Primary outcome is 16 weeks post randomisation but measurement at 52 weeks will advise if early evidence for effectiveness is sustained, answering important questions about cost-effectiveness.
- Measurement at 52 weeks may also reduce potential bias caused by lengthy waiting lists in some UK health service regions for the comparator intervention that is, ‘NHS support for depression’.
- The diversity of trial centres improves the chances of recruiting a representative cohort of participants and involvement of practitioners at these centres the generalisability of findings.
- Participants are not blinded to their allocation, which may affect responses on the self-report primary outcome measure.

## Introduction

### Background

 High rates of mental health conditions co-occur with autism, particularly common mental health problems such as anxiety and depression. Depression is three to four times more prevalent in autistic people^1^ than the general population, and is associated with reduced quality of life.^2^ A total population study of 223 842 individuals in Stockholm County reported that, of the 4073 who had an autism diagnosis, 19.8% had also been diagnosed with depression by the age of 27 years, compared with 6% of the general population (adjusted risk ratio 3.6).^3^ A meta-analysis of adult autism studies (n=26 070 participants in 29 studies, 17 UK-based) reported pooled estimates of current and lifetime prevalence of depression to be 23% and 37%, respectively.^4^ These figures are in stark contrast to the 3–4% current point prevalence of depression in the UK general population.^5^

Effective treatments for depression exist. UK National Institute for Health and Care Excellence (NICE) guidelines^6^ recommend low-intensity psychosocial interventions based on the principles of cognitive-behavioural therapy (CBT) as an evidence-based treatment for mild-moderate depression.

Even though autistic people have positive views towards participating in randomised controlled trials (RCTs),^7^ they are under-represented in, if not explicitly excluded from such studies.^8^ Clinical guidelines about the treatment of depression are then based on research evidence that may not include autistic people. Furthermore, depression can present atypically in autistic people^9^ highlighting the relevance of autism-specific research.

The provision of effective empirically supported treatments for depression is a priority for the autistic community and healthcare services (https://www.autistica.org.uk/our-research/our-research/your-research-priorities). The National Health Service (NHS) England long-term plan (2019) (www.longermplan.nhs.uk) cites improving healthcare services for autistic people as an NHS priority, including community mental health support and suicide prevention.

A recent retrospective matched observational study linking UK electronic healthcare records with NHS talking therapies for anxiety and depression service data considered the outcomes of >8000 adult attendees with an autism diagnosis across a 7-year period.^10^ Moderate pre–post therapy effect sizes were reported for depression and anxiety for the autism group with reliable improvement and recovery rates (56.2% and 31%, respectively) promising but slightly lower (68.2% and 46.4%) when compared with adults without a diagnosis of autism. These findings highlight the need to develop an evidence base and improve treatment outcomes for autistic people experiencing depression.

There is evidence that CBT can be effective in treating anxiety if adapted to meet the needs of autistic people.^11^ Differences in social communication, neurocognition and emotional awareness in autism^12^ underpin the need to adapt psychosocial treatments. A recent study provides evidence of an advantage of adapted CBT over standard CBT for anxiety in autistic young people.^13^ However, there have been no definitive treatment evaluations of adapted CBT approaches for depression for autistic adults. A meta-analysis of cognitive behavioural interventions in autism^11^ identified two small studies of depression treatment meeting inclusion criteria: mindfulness-based stress reduction for adults^14^ and combined anxiety and depression group CBT for adolescents.^15^ There has since been a study of combined anxiety and depression CBT in adults (N=59),^16^ and two non-randomised studies of adapted group CBT for depression in adolescents.^17 18^ Our recent feasibility study^19 20^ reported positive changes in depression scores. A recent study investigating dialectial behaviour therapy (DBT) for suicidal ideation and behaviour reported a reduction in depression severity for the DBT group.^21^ Taken together, these studies provide preliminary evidence that adapted CBT may be helpful for depression, but the findings need confirmation in a definitive trial.

In an earlier pilot RCT we demonstrated the feasibility of developing and delivering a low-intensity intervention (guided self-help; GSH) for depression based on behavioural activation (BA) adapted for the needs of autistic adults.^19 20^ The intervention (GSH) comprised materials for nine individual sessions facilitated by a low-intensity psychological therapist who received training and an accompanying manual. It was possible to recruit the target number of participants (n=70) on time for the study. Rates of withdrawal from the GSH arm of the study were low (9%), retention at 16 weeks was high (86%) suggesting the research design with randomisation was acceptable. The rate of withdrawal from the treatment as usual (TAU) arm was 17% and retention at 16 weeks was poor (54%). The GSH was well-received by participants and therapists; 86% of participants attended the predefined ‘dose’ of six treatment sessions and 71% attended all nine sessions. We used two self-report (Patient Health Questionnaire-9 (PHQ-9) and Beck Depression Inventory-II (BDI-II)) and one interview measure (Hamilton Rating Scale for Depression^22^ of depression in the feasibility study. Inter-rater reliability for the interview measure was less than adequate, the two self-report measures were well-aligned and many participants suggested a preference for the BDI-II as a self-report measure with item sets of closed statements less subject to misinterpretation. The findings indicated the GSH intervention was promising and acceptable. The clinical effectiveness and cost-effectiveness of this intervention in a large-scale RCT is now warranted.

The primary aim of the ADEPT-2 trial is to establish the clinical and cost-effectiveness of an adapted low-intensity psychological intervention (GSH) for treating depression in autistic adults when compared with TAU. TAU is the comparator to enable full evaluation of cost-effectiveness and to inform policymakers and commissioners of UK NHS services about the most effective treatment for depression for autistic adults. The impact of treatment (GSH vs TAU) on the carers of participants taking part in the trial will also be explored in the carer substudy. The ADEPT-2 trial also incorporates qualitative work to explore participants’ and therapists’ acceptability, experiences of and adherence to, treatment principles. This is the first full-scale RCT of an evidence-based low-intensity psychological intervention for depression adapted for autism.

### Study Within A Trial

A Study Within A Trial to inform the design of interventional RCTs for autistic adults with a treatment-as-usual condition is implemented as a separate substudy.^23^

## Methods and analysis

### Trial design

A two parallel-group multicentre pragmatic RCT comparing GSH for depression with TAU. The trial methods were informed by the design of the earlier feasibility study with several significant changes.^20^ Following a comparison of potential measures in the feasibility study,^20^ the BDI-II^24^ score at 16 weeks post randomisation was selected as the primary outcome. Participants in the feasibility study also expressed a preference for the format of the BDI-II over the PHQ-9.^20^ Follow-up continues until 52 weeks and reimbursement for completion of follow-up measures is used to address issues of differential attrition from the TAU arm during the feasibility study. Participants are offered a £10.00 gift voucher to thank them for their time after the completion of each of the four questionnaires.

### Setting

ADEPT-2 will be delivered across six regional centres in England and Wales:

1. Southwest England—Avon and Wiltshire Mental Health Partnership NHS Trust.
2. North of England—Cumbria, Northumberland, Tyne and Wear NHS Foundation Trust Wear.
3. Northeast of England—Tees, Esk and Wear Valleys NHS Foundation Trust.
4. East Midlands—Leicestershire Partnership NHS Trust.
5. West Midlands—Coventry and Warwickshire Partnership NHS Trust.
6. Wales—Cardiff & Vale University Health Board.

Sites 1 and 2 were involved in the feasibility study (ADEPT-1) and average monthly recruitment of potentially eligible participants by these sites informed the design of the present trial. Sites 3–6 were approached to increase diversity in recruitment by involving sites with areas of high urban populations as well as sites where autism research is less represented.

Outside of these regional centres, potentially eligible participants from any region in England and Wales can participate in the trial.

The majority of trial appointments will be conducted via Sponsor/NHS-approved video-conferencing. Alternative methods of communication (eg, face-to-face visits, and/or other remote methods) will be considered and facilitated, where feasible and preferred by participants.

### Trial population

Adults with a clinical diagnosis of autism and symptoms of depression who would consider a low-intensity psychological intervention (GSH) for depression.

People will be eligible to take part if all of the following apply:

- Adult aged ≥18 years.
- A clinical diagnosis of autism spectrum disorder.
- Current depression measured by the PHQ-9 with a score of ≥10 at screening informed by validity studies of the PHQ-9 and to align with routine UK NHS care.
- Can be on medication but the dose should be stable for 6 weeks prior to randomisation.

Patients will be excluded from the trial if any of the following apply:

- Risk of suicide or severity of depression such that a low-intensity psychological intervention is not clinically indicated, as judged by the site lead clinical researcher. Participants who endorse a score of 3 on item 9 of the PHQ-9 will be followed-up to assess suicide risk.
- Individual psychological treatment (>6 sessions) within a cognitive behavioural framework during the previous 6 months.
- A history of psychosis.
- Current alcohol/substance dependence.
- Untreated epilepsy.
- English and Welsh literacy levels such that the treatment materials are inaccessible without reasonable adjustments, and a supporting person is not available. We will strive to include all adults in the study if supporters are available to help an individual access the treatment where written/spoken English, non-English and Welsh presents a barrier.
- Co-enrolment in other potentially competing (ie, mental health) interventional research studies.

### Patient approach and consent

A range of recruitment pathways will ensure that the adult autism population is fully represented in this trial: (1) clinical appointments and/or clinical lists (eg, autism diagnostic services); (2) research registers/cohorts/charities; (3) self-referrals and other methods (eg, recruitment materials displayed in relevant locations or promoted via social media).

When potentially eligible participants are identified they will be sent an invitation letter and participant information leaflet. Alternatively, they may be directed to the trial website containing equivalent documentation for online review if preferred. Potential participants will then be directed to complete an expression of interest form (EOI; online or paper). The EOI form can be completed by the individual and/or the healthcare professional referring them to the trial. The form will ask a series of questions based on broad inclusion/exclusion criteria, including the items from the PHQ-9.

Potentially eligible individuals will be invited to attend an appointment with a suitable participating site during which the local researcher will answer any questions, confirm eligibility criteria, receive written informed econsent (if the individual decides to take part) and complete any outstanding baseline data collection. Potentially eligible individuals known to healthcare services at site, that is, with a current electronic healthcare record, will be asked to give permission for professionals involved in their care to be contacted for further consideration of suitability of taking part in the study in the context of broader service delivery.

The Prinicipal Investigator (PI) reviews all information gathered at baseline and makes the final decision regarding participant eligibility and randomisation. Participants will be asked if they have a carer and, with the participant’s agreement, the carer will be approached for their consent to take part in the carer substudy. The ADEPT-2 participant consent form can be seen in appendix 1).

### Trial intervention/randomised treatments

#### Guided self-help

The GSH intervention is based on the principles of BA, the recommended treatment model for low-intensity CBT for depression. BA^24^ encourages people to become more aware of the range of behaviours and situations associated with different moods and use this information to make changes in line with their individual goals through activity scheduling. Doing more of what makes you feel good is the key mechanism.

Participants randomised to GSH are provided with a booklet (electronic and print/pdf) containing informational materials for nine topics. They are invited to attend nine individual meetings with a therapist guide (hereon in called a GSH coach) ordinarily held at weekly intervals. GSH sessions can last up to 45 min (except for the first session which can last up to 90 min). Sessions are held online using the NHS-approved platform for that service/region. If participant preference is for in-person attendance, where feasible this will be facilitated. During GSH sessions, the materials are reviewed and discussed on screen with collaborative work and annotation to encourage personalisation of the electronic and/or print versions of the booklet according to individual preference. Between session tasks are suggested to consolidate the treatment principles, and this is checked for completion and quality by the coach at the next appointment.

GSH coaches are ordinarily graduate-level psychological practitioners or other mental health professionals with foundation knowledge of cognitive behavioural principles. GSH coaches receive 15 hours of training and an accompanying coach manual. GSH coaches attend weekly group supervision.

The GSH intervention was developed for the feasibility study and refined on the basis of participant feedback. Additional visual images were commissioned from an autistic graphic artist to improve the accessibility and appearance of the materials.

Each GSH session covers a key principle(s) which are delivered in a developmental sequence to consolidate learning. The first session is an orientation session to introduce GSH and understand an individual’s needs in respect of autism. Sessions 2–4 take a focus on noticing situations, different behaviours in situations, granularity of behaviours and rating positive feelings. Session 5 introduces activity scheduling, Session 6 the concept of meeting different levels of need, Sessions 7 and 8 consolidate and expand on activity scheduling and Session 9 comprises review, reflect and planning ahead.

The intervention is described in the feasibility study outcome paper.^20^

Consistent with low-intensity treatment recommendations, depression and anxiety symptoms are monitored weekly using the PHQ-9 and Generalised Anxiety Disorder Assessment (GAD-7).

GSH should commence 2 weeks post randomisation subject to individual availability of participant and coach. Attendance at ≥6 sessions of GSH will be considered an adequate treatment ‘dose’ as the main treatment principles have been introduced.

Delivery of the GSH intervention will be monitored by participants’ and GSH coaches’ recorded therapeutic alliance, using client and therapist versions of the Working Alliance Inventory-Short Revised (WAI-SR).^25^ This will be completed by GSH coaches and participants once within the first four sessions and once within the second four sessions at the same time as goal attainment measurement. Adherence to GSH content will be measured through a scale specific to the manual identifying key elements of therapy for each session. Therapist coaches will complete a self-rating of adherence to content for each session using this scale. Two GSH sessions, randomly allocated, will be audio recorded. 20% of these recordings will be randomly sampled to validate the therapist self-rating of adherence. ‘Intervention receipt’ will be monitored through therapist records of participants’ completed exercises and homework activity, ease of delivery and client engagement with the materials.

#### Treatment as usual

There are no constraints on TAU. Participants randomised to TAU are signposted to NHS talking therapy services for anxiety and depression. Randomisation to TAU is communicated to participants’ general practitioner (GP) by letter and the range of treatment options can be considered by an individual with their GP.

#### Intervention withdrawal

Participants can choose to withdraw for any reason at any time during their involvement in the trial. For participants receiving the GSH intervention, if there is evidence of increased risk of suicide and/or worsening of mental state as evidenced by session-by-session administration of the PHQ-9, the PI or suitably trained staff member can decide to withdraw participants based on clinical opinion. Referral to statutory mental health services will be discussed with the participant and recommended to the GP with clinical responsibility.

#### Randomisation

Randomisation will be performed after eligibility is confirmed by the site principal investigator (or authorised delegate), informed consent has been obtained and baseline assessments have been completed.

Patients will be allocated in a 1:1 ratio to GSH or TAU. The randomisation sequence will be generated by Sealed Envelope^26^ stratified by centre, depression severity as captured by baseline BDI-II score (0–25, 26–35, 36–63), and current prescription of anti-depressant medication (yes/no). A participant’s allocation will only be revealed to the site once they are added to the trial Research Electronic Data Capture (REDCap) database and their baseline data has been entered.

### Blinding

The Trial Management Group (TMG) will be blinded to the allocation of treatment group, except for the trial statistician, trial manager and data manager. Two statisticians will support this trial: the trial statistician who will report unblinded analyses to the data monitoring committee and the supervisory statistician who will remain blinded while recruitment and follow-up are ongoing. Clinicians (PIs), other researchers and site staff will not be blinded.

Participant unblinding is only required if information about allocation would affect the clinical response to a crisis such as the risk of suicide. Unblinding will be carried out by the central trial management team or local Research Assistant (RA) in such situations.

#### Primary and secondary outcome

The primary outcome for this trial is BDI-II^27^ score at 16 weeks post randomisation as a continuous outcome. The BDI-II is a 21-item self-report measure of depression and has been evaluated in terms of psychometric properties for use with autistic adults.^28^ A further advantage in using the BDI-II is conferred as it is not the routine outcome measure in UK NHS talking therapy services for depression and is less subject to repeated administration outside of the trial.

The secondary outcomes include (see table 1):

1. Depression symptoms using:
  1. BDI-II^29^ depression score measured at 32 and 52 weeks post randomisation.
  2. PHQ-9^30^ measured at 16 and 52 weeks post randomisation. The PHQ-9 is a nine-item self-report measure of depression.
  3. Global Rating of Change^31^ is a single self-assessed 5-point scale of change in a specific condition (depression) measured at 16, 32 and 52 weeks post randomisation.
2. Quality of life using the EuroQol five-dimension health status questionnaire (EQ-5D-5L) and EuroQol-Visal Analogue Scale (EQ-VAS)^32^ measured at 16, 32 and 52 weeks post randomisation.
3. Anxiety using the GAD-7 questionnaire^33^ a seven-item self-report measure of anxiety measured at 16 and 52 weeks post randomisation.
4. Positive and negative affect using the Positive And Negative Affect Schedule (PANAS).^34^ The PANAS is a 20-item self-report measure of positive and negative affect 16 and 52 weeks post randomisation.
5. Work and social function using the Work and Social Adjustment Scale^35^ a five-item self-report measure of impaired functioning measured at 16 and 52 weeks post randomisation.
6. Carer impact using:
  1. Depression Anxiety Stress Scales^36^ a 42-item self-report measure of three related negative emotional states of depression, anxiety and tension/stress measured at 16 and 52 weeks post randomisation.
  2. Warwick-Edinburgh Mental Well-Being Scale^37^ a 14-item self-report measure of mental well-being focusing on positive aspects of mental health measured at 16 and 52 weeks post randomisation.
7. Resource-use via a participant-reported resource-use questionnaire, including ModRUM (^38^ the Work Productivity and Activity Impairment: General Health (WPAI-GH) and bespoke questions measured at 16, 32 and 52 weeks post randomisation. The ADEPT-2 ModRUM is 14 items, and includes the ModRUM core module, questions capturing NHS counselling or any other ‘talking therapy’ and prescribed medications. Five bespoke items cover social care, voluntary services and personal expenses. The WPAI-GH^39^ is a six-item self-report measure of societal productivity losses.
8. Cost-effectiveness via quality-adjusted life years (QALYs), generated from the EQ-5D-5L and resource use questionnaire measured at 16, 32 and 52 weeks post randomisation.

**Table 1:** Trial assessments and key participant-related procedures

Data collection time point (→)	Pre-randomisation	Point of randomisation	Post randomisation				
Key data capture (measures)/trial procedures (↓)	Baseline	Baseline	1–15 weeks		16weeks*	32weeks	52weeks
Screening	●						
Eligibility assessment	●	●					
Consent to join trial and randomisation		●					
Demographics		●					
Revised clinical interview schedule		●					
Beck Depression Inventory-II (primary outcome at 16 weeks)		●			●	●	●
Generalised Anxiety Disorder Assessment		●			●		●
Patient Health Questionnaire-9 (depression symptoms)	●	●			●		●
Work and Social Adjustment Scale		●			●		●
Self-rating of global change					●	●	●
Positive And Negative Affect (PANAS-SF)		●			●		●
Health-related quality of life (EQ-5D-5L and EQ-VAS)		●			●	●	●
Work Productivity and Activity Impairment Questionnaire: General Health		●			●	●	●
Health and social care resource use questions					●	●	●
Guided self-help sessions*							
Case report form(s) - therapist records							
Working Alliance Inventory (Short Revised)* (therapist and participant rated)			●	●			
Goal attainment record*			●	●			
Qualitative interviews with patients and therapists†					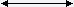		
Carer substudy: Depression Anxiety Stress Scales and Warwick-Edinburgh Mental Well-Being Scale		◊			◊		◊

Key: ● data capture / outcome measures; completion methods may vary depending on participant preferences. **◊** completed by caregiver.

* Intervention arm only.

† Months 6–12 decline/withdrawal qualitative interviews, months 10–20 end of treatment GSH participant qualitative interviews, 16–24 months TAU participant qualitative Interviews.

EQ-5D-5LEuroQol five-dimension health status questionnaire EQ-VASEuroQol Visual Analogue ScaleGSHguided self-helpPANAS-SFPositive And Negative Affect Schedule-Short FormTAUtreatment as usual

### Provisions for post-trial care

Participants’ GP are informed about the end of trial participation at 52 weeks. Any indication of worsening depression symptoms, increased risk or need for a higher level of clinical care prompted by participant response at outcome measurement is communicated to the GP as per risk protocol. This applies at 52 weeks.

### Data collection

Participants will be asked to complete an ADEPT-2 follow-up questionnaire at 16, 32 and 52 weeks post randomisation. Participants will be asked to complete the questionnaires online and will receive a secure online link at the appropriate time points. Alternative methods preferred by the participant will be considered and facilitated where feasible (eg, by video call (using Sponsor/NHS-approved video-conferencing tools), postal hard copy, face-to-face or telephone). If the participant requires assistance to complete the questionnaires, the research team will aim to try and make all reasonable adjustments requested by the participant to facilitate this. Similarly, a carer/family member or friend can provide support, but they will be advised not to answer any questions on behalf of the participant.

Carers taking part in the carer substudy will be asked to complete online follow-up questionnaires at 16 and 52 weeks after the participant they care for was randomised in the main trial.

### Data management

Data from all participants will be collected and retained in accordance with the UK Data Protection Act 2018 and the UK General Data Protection Regulation 2018 (GDPR). All trial participants will be allocated a unique study ID number during the screening process, which will remain assigned to them. Data from all participants will be captured electronically via REDCap. However, where electronic data collection is not possible, equivalent paper documents will become the source data. Personal identifiers will be kept in a secure database that is only accessible from within the UniBristol firewall. Anonymised clinical data will be held on a separate server and will be linked by a participant ID.

The University of Bristol and University of Bath are joint data controllers for the ADEPT-2 trial. Data will be held at the University of Bristol and will conform to the University of Bristol Data Security Policy and in compliance with the UK GDPR, alongside the Data Protection Act 2018. Secure email links, econsent, automated reminders.

Data will be retained for at least 5 years after the end of the trial, and at the end of the archiving period, will be destroyed by confidential means with the exception of a final data set which will be made available for data-sharing purposes.

### Sample size

99 participants in each of the GSH and TAU groups and returning the primary outcome measure will allow a minimum clinically important difference of 0.4 SD^40^ on the primary outcome (approximately four points on the BDI-II scale, a 13% reduction on the mean baseline score of 31 seen in the external pilot study participants) to be detected with 90% power at the 5% significance level, in an analysis adjusting for the baseline measure of the primary outcome and assuming a correlation of 0.5 between baseline and 16-week assessments. We set a sample size target of 248 participants (124 in each group) to allow 90% power to be achieved with up to 20% of primary outcome measurements being missing.

### Statistical analysis

A detailed statistical analysis plan will be written prior to the trial data being released for analysis and will be made publicly available. The primary analysis will follow the intention-to-treat analysis principle, comparing BDI-II responses at the 16-week assessment point between the groups as allocated. The treatment effect on the primary outcome will be estimated by a linear regression model with covariates including the baseline BDI-II measure, trial centre, current prescription of antidepressant medication and treatment group allocation. The treatment effect will be estimated as the coefficient of the treatment group allocation covariate, with a 95% CI and p value. This approach will be adapted to the secondary measures. Sensitivity analyses will explore the potential impact of any missing primary outcome data. Any subgroup analyses will be prespecified in the statistical analysis plan. No interim analyses are planned.

To investigate the correlation between attending sessions and the primary outcome response, we will present summary statistics for the 16-week BDI-II for the intervention group participants who attend 0, 1–5 and 6 or more sessions. A sensitivity analysis will repeat the primary analysis on a complier average causal effect basis, comparing intervention group participants attending one or more sessions against the estimated outcome of the comparable participants allocated to the comparison group (ie, those comparison group participants who would have attended one or more sessions had they instead been allocated to the intervention).

### Health economic analysis

The primary cost-utility analysis will assess the cost-effectiveness of GSH compared with TAU at 52 weeks, from a societal perspective including the NHS, personal social services (PSS), personal expenses, voluntary services and productivity. A secondary analysis will restrict the perspective to that of the NHS and PSS to conform to the NICE reference case.^41^ Utility values will be estimated from EQ-5D-5L scores collected at baseline, 16, 32 and 52 weeks follow-up using the NICE-recommended approach at the time of analysis. QALYs will be estimated from utility scores using the area under the curve approach, adjusting for baseline utility.^42^

Intervention costs (including training, delivery and supervision) will be recorded in study records. All-cause resource use, including primary, community and secondary care, prescribed and over-the-counter medications, time off paid employment, social care contacts, travel for healthcare and charity support services, will be captured via participant-report at 16, 32 and 52 weeks follow-up.^38 43^ Resources will be valued using published unit costs for the most recent cost year available at the time of analysis.^44^,^47^ Given the 1-year study duration, discounting will not be conducted.

A predefined health economics analysis plan to guide analysis will be prepared and made publicly available. Missing data patterns will be reviewed and handled appropriately. Net monetary benefit statistics, and if appropriate incremental cost-effectiveness ratios, will be calculated to assess cost-effectiveness. Uncertainty will be explored through cost-effectiveness acceptability curves and one-way sensitivity analyses.

### Qualitative study

To examine the views and experiences of the intervention and the trial, we will conduct in-depth qualitative interviews with up to 60 trial participants and 10 GSH coaches. Topic guides will be used for all interviews, specific to those being interviewed, which can be found in the online supplemental information. Qualitative findings will identify factors that may impact on the intervention acceptability and effectiveness. All participants in the trial will be asked if they are willing to be contacted about taking part in an interview at the time of trial consent. Autistic adults who decline or withdraw from participation in the main trial will be approached as soon as possible about taking part in an interview. Trial participants will be approached about an interview after they have completed their 16-week follow-up questionnaire, and the therapists will be approached at 4 months. The qualitative researcher will contact the participants via the participants’ preferred contact method and confirm if they would like to take part in the interviews. All interviews will be conducted remotely (by telephone/video call) and with informed consent, interviews will be audio recorded. Purposive theoretical sampling will ensure diversity in demographic characteristics (eg, age; gender; ethnicity; and socioeconomic status). Sample size will be determined by the concept of ‘information power’,^48^ with continuous assessment of information within our sample regarding meeting trial objectives. Data will be analysed using a thematic approach,^49^ and will be conducted in parallel to data collection, with findings from early analysis informing later data collection in an iterative process.

### Trial management and oversight

The Bristol Trials Centre will be responsible for the day-to-day management of the trial, including the preparation of trial documents, training and monitoring of centres. The TMG, a core working group of staff, will oversee the trial and meet regularly to review milestones. The TMG report to the Trial Steering Committee (TSC). The TSC are an independent committee that make recommendations and key decisions during the trial. The Data Monitoring Committee (DMC) are an independent committee that assess the safety and efficacy of the trial’s interventions, monitor the trial’s overall conduct and protect its validity and credibility. Membership of the trial oversight committees is described in the Acknowledgements section.

### Safety

In accordance with Good Clinical Practice guidelines, adverse events and risk standardised operating procedures will be followed by all researchers and GSH coaches working on the trial. Adverse event data will be collected for the duration from randomisation to 52 weeks post randomisation. Adverse events will be identified during GSH appointments for participants allocated to GSH up to the 16-week follow-up, where adverse events will be detected via trial questionnaires (across GSH and TAU). The central and/or local research team will report any Adverse Events (AEs) that occur, should they become aware. The PI of each participating site is responsible for assessing all adverse events and categorising whether they are serious, expected and related. All suspected unexpected serious adverse reaction will be reported to the Sponsor, Research Ethics Committee (REC) and DMC.

### Auditing

The trial will be monitored and audited in accordance with the Sponsor’s policy, which is consistent with the UK Policy Framework for Health and Social Care Research.

### Patient and public involvement

A patient advisory group comprising autistic adults, carers and a CBT therapist with lived experience support the trial throughout. This will include attending trial management and steering committees as experts by experience to consult the research team, review of the trial documentation and protocol and development of communication strategies to support recruitment and dissemination activities.

### Major protocol amendments

The current protocol is V.3.0, 12 August 2024. The key change from V.1.0 was the addition of a telephone call 2 weeks post randomisation for TAU participants and the collection of information about possible Attention Deficit Hyperactivity Disorder (ADHD) and presence of an intellectual disability at baseline to characterise the sample further. The key change from V.2.0 was to clarify and correct errors in table 3, and to update the study timelines in line with a contract extension. The full protocol is available from the National Institute for Health and Care Research (NIHR) Journals web page (https://www.fundingawards.nihr.ac.uk/award/NIHR132343).

## Ethics and dissemination

The trial received REC approval from East of England—Essex REC and Health Research Authority approval in June 2022. The trial is hosted by Avon and Wiltshire Mental Health Partnership NHS Trust, is sponsored by the University of Bath and is coordinated by the Bristol Trials centre, a UK Clinical Research Collaboration registered trials unit.

On completion of the trial, a final report will be prepared for the Funder (NIHR Health Technology Assessment). The findings will be submitted for publication in relevant academic journals. An accessible summary of the findings will be disseminated to participants and made publicly available.

### Access to the final data set

Anonymous research will be stored securely and kept for future analysis with participant consent. We anticipate that anonymised trial data will be shared with other researchers to enable prospective meta-analyses. Data will be kept anonymous in a research data storage facility (RDSF). Requests for access to data must be via a written confidentiality and data sharing agreement available from the RDSF website which will be confirmed by the Chief Investigator (CI) (or appointed nominee).

### Trial progress

Recruitment started on 15 August 2022 and is due to be complete by 29 February 2024. An internal pilot finished in January 2023 and the funder gave approval for the trial to move into the main phase of recruitment, which is ongoing.

## supplementary material

10.1136/bmjopen-2024-084729 online supplemental file 1

10.1136/bmjopen-2024-084729 online supplemental file 2

10.1136/bmjopen-2024-084729 online supplemental file 3

10.1136/bmjopen-2024-084729 online supplemental appendix 1
