# Supplementary material for: Effectiveness and cost-effectiveness of guided self-help for depression for autistic adults: the Autism Depression Trial (ADEPT-2) – protocol for a multicentre, randomised controlled trial of a remotely delivered low-intensity intervention
Source: BMJ Open. 2024 Nov 19;14(11):e084729. doi: 10.1136/bmjopen-2024-084729 (PMC11580278; doi:10.1136/bmjopen-2024-084729)
Supplement: online supplemental file 1 [file bmjopen-14-11-s001.docx]

**Part A. Introduction, consent and background**

Thanks, introduce self, re-state purpose of the interview

- Discussion of how interview will be recorded, right to withdrawal, issues of confidentiality, anonymisation and informed consent. (*written consent to follow if not obtained already).* Verbal consent (if no written consent yet): *switch* *audio recorder on* - For the audio recording, can I check that:
- You read and understood the study information sheet?
- You know that taking part in the interview is voluntary and you are free to stop the interview at any point and you may skip questions you would prefer not to answer?
- You agree to our conversation being audio recorded?
- You understand that quotes from the interview may be used to illustrate our findings but it will not be possible to trace who said them?
- Background information on participant (e.g. age, ethnicity, location, general health)
- Previous experience of therapy

**Part C: Reasons for declining/withdrawing**

- Why did you decide not to take part in the study/withdfraw from study?
- Were they any other reasons?
- Did you discuss your decision with others? If not, did you want to?
- Have you taken part in a research study before? If so, what were their experiences of doing so?

## **Part D: Concluding the interview**

- Is there anything you would change about the study that may make you more interested in taking part?
- Can you think of anything that we can do to encourage people to take part in the study?
- Is there anything else you want to say about the study?

**Final thoughts**

- Thank you all so much for your discussion. Do you have any final points that you would like to discuss or that you feel you didn’t have the opportunity to say?

**Part B: Approach and information received**

- Can you remember how you heard about the study?
- How did you find the study information?
- What was your understanding about the aims of the study?
- What was your understanding what taking part in the study would have involved?
  - Randomisation, equipoise, arms
- How did you feel about being asked to take part in a study ?

**Notes**
