# Supplementary material for: Effectiveness and cost-effectiveness of guided self-help for depression for autistic adults: the Autism Depression Trial (ADEPT-2) – protocol for a multicentre, randomised controlled trial of a remotely delivered low-intensity intervention
Source: BMJ Open. 2024 Nov 19;14(11):e084729. doi: 10.1136/bmjopen-2024-084729 (PMC11580278; doi:10.1136/bmjopen-2024-084729)
Supplement: online supplemental file 2 [file bmjopen-14-11-s002.docx]

**Part B. Trial views**

- Can you remember how you heard about the study ?
- How did you find the study information?
- How did you feel about being asked to take part in a study?
- Trial understanding
  - aims of the study?
  - what taking part in the study would involved? expectations
  - terms: randomisation, equipoise
  - guided self-help – purpose, details.
  - Usual care – purpose, details.
  - Do you recall reading about training resources about working with autistic people being available to local services? Did this information weight in on your decision to take part?
- preference for arm Why did you decide to take part? Did you discuss your decision with others?
- What were your expectations of taking part in the study?
- Acceptability/experience: What has it been like to take part in this study?
- What do you think has worked well?
- Challenges/what could have improved experience of taking part?
- Suggested improvements
- Anything that you would have liked to have received more information about?

**Part A. Introduction, consent and background**

Thanks, introduce self, re-state purpose of the interview

- Discussion of how interview will be recorded, right to withdrawal, issues of confidentiality, anonymisation and informed consent. (*written consent to follow up if not obtained, telephone verbal consent).* Verbal consent: *switch* *audio recorder on* - For the audio recording, can I check that:
- You read and understood the study information sheet?
- You know that taking part in the interview is voluntary and you are free to stop the interview at any point and you may skip questions you would prefer not to answer?
- You agree to our conversation being audio recorded?
- You understand that quotes from the interview may be used to illustrate our findings but it will not be possible to trace who said them?
- Background information on participant (e.g. age, ethnicity, location, general health)
- Previous experience of therapy

**Part C: Experience of trial participation**

**Intervention arm**

- Views on guided self help, understanding of aims of self help ? expectations? acceptability ?
- Personal goals – what they wanted to get out of it / were goals met
- Self-efficacy – did they want to and feel able to help themselves with their low mood?
- Self help material: understandable/level, format (paper/online), pace, read the materials between sessions/ever return to the materials?
- The map – was it necessary? how was it used (i.e. as given, with own photos)?
- Feelings chart – did they need and use a visual chart?
- In session 1, what was your experience of task 2: What does my coach need to know about you autism?
- Sessions - best/worst, why ?
- Was the amount of content each session about right, too much, too little?
- Sessions: views on number, length of time, frequency of sessions suitable ?
- Between session activities/homework: which most helpful, why, changes made, facilitators/barriers, how overcome barriers, improvements
- Therapist/guide: understanding of your autism, autism knowledge helpful, what did to help, engagement, support level (understand the nature of a coach), needed for self help?
- Did they feel connected with and supported by others?
- What worked well, what was useful about the treatment?
- What 2 things have you learnt as a result of the treatment?
- Did the participant understand the principles of the treatment?
- Suggested improvements: name 2 things that could be better/improved?

**Usual care arm**

- Describe treatment received – F2F, group, telephone? Howuseful? Acceptabile?
- Personal goals? Were these met?
- If psychological intervention - understanding of aim, Therapist: understanding of your autism,enough level of support? Ability to communicate with autistic people?
- Did they feel connected with and supported by others?
- Self-efficacy – did they want to and feel able to help themselves with their low mood?
- What worked well, what was useful about the treatment?
- Suggested improvements, name 2 things that could be better/improved?

**Final thoughts**

- Would it be helpful to have someone else (friend/family) present in therapy
- Personal changes: mood/depression/activties/relationships - facilitators/barriers
- Questionnaire: how did you find filling out the questionnaires? usefulness/if filling out? Explore which measure of depression best captured experience/could best relate to
- Is there anything that we have not talked about that you would like to raise?
- Would you like us to send you a brief report of the study’s findings?
- How would you like us to send you the voucher? Postal or online ? Thank them for their time
