# Supplementary material for: Effectiveness and cost-effectiveness of guided self-help for depression for autistic adults: the Autism Depression Trial (ADEPT-2) – protocol for a multicentre, randomised controlled trial of a remotely delivered low-intensity intervention
Source: BMJ Open. 2024 Nov 19;14(11):e084729. doi: 10.1136/bmjopen-2024-084729 (PMC11580278; doi:10.1136/bmjopen-2024-084729)
Supplement: online supplemental file 3 [file bmjopen-14-11-s003.docx]

**Part C: Guided self help**

- Views on self help - therapeutic principals appropriate for this client group/ appropriate treatment model
- Could you identify which aspects of guided self-help are most important for the clients?
- Views on Format, Content, deliverability
- Sessions: best/worst, why ?, content too much/little, views on number, length of time,
- Pace and order of the sessions – did you do the sessions in order? Flexibility?
- Self help material: engagement (e.g. map, feeling chart), understandable/level, formatt (paper/online), pace
- Between session activities: which most/least successful, why, facilitators/barriers, how overcome barriers, improvements
- What aspects did patients need most/least help
- Reaction from clients regarding guided self-help (with some concrete examples)
  - understanding treatment aims, goals
  - engagement with tasks set between sessions
  - Other feedback to the therapist
- Suggested improvements: name 2 things that could be better/improved?

**Final thoughts**

- In a bigger trial, would you make any other changes to the trial design?
- If guided self-help was to show a benefit for depression in adults with autism spectrum disorders do you think that there would be any issues with it being taken up as part of standard care? Why? Implications?
- Are there any changes that would need to be implemented for it to be rolled out to standard care?
- Are there any other changes that you could suggest?
- Is there anything that we have not talked about that you would like to raise?
- Would you like us to send you a brief report of the study’s findings?
- Thank them for their time

**Notes**

**Part B. Trial impression and experiences**

- How did you first hear about the ADEPT study? First impressions?
- When ADEPT first started we were under the impression that both approaches [guided self help Vs ususal care] were equally good? Why? Have your views about this changed since starting? Why?
- Experience: What has it been like to take part in this study?
- Involvement in ADEPT? [number of patients]
- Do you think the trial has been successful?
- Overall, what do you think has worked well with the trial?
- Challenges/what could have improved experience of taking part?
- Anything that you would have liked to have received more information about?
- Suggested improvements to trial design
- How did you feel providing therapy for the client group? previous experiences, challenges, training needs

**Part A. Introduction, consent and background**

- Thanks, introduce self, re-state purpose of the interview
- Discussion of how interview will be recorded, right to withdrawal, issues of confidentiality, anonymisation and informed consent. (*written consent to follow if not obtained, telephone verbal consent).* Verbal consent: *switch* *audio recorder on* - For the audio recording, can I check that:
- You read and understood the study information sheet?
- You know that taking part in the interview is voluntary and you are free to stop the interview at any point and you may skip questions you would prefer not to answer?
- You agree to our conversation being audio recorded?
- You understand that quotes from the interview may be used to illustrate our findings but it will not be possible to trace who said them?
- Background information on participant (e.g. age, ethnicity, lenth of time since qualifaction, experience working with adults with autism spectrum disorders)
