# Supplementary material for: Effectiveness and cost-effectiveness of guided self-help for depression for autistic adults: the Autism Depression Trial (ADEPT-2) – protocol for a multicentre, randomised controlled trial of a remotely delivered low-intensity intervention
Source: BMJ Open. 2024 Nov 19;14(11):e084729. doi: 10.1136/bmjopen-2024-084729 (PMC11580278; doi:10.1136/bmjopen-2024-084729)
Supplement: online supplemental appendix 1 [file bmjopen-14-11-s004.pdf]

Centre for Applied Autism Research (CAAR) | UNIVERSITY OF BATH | BRISTOL TRIALS CENTRE

[site logo]

ADEPT-2
Autism Depression Trial

IRAS ID: 310263

Centre Number:__________

Study Number: ___________

Participant Identification Number for this trial: ______________

ADEPT-2 STUDY CONSENT FORM

Title of Project: Autism Depression Trial- 2 (ADEPT-2)

Name of Researcher:__________________________

Please initial box

1. I confirm that I have read the information sheet dated................... (version...........) for the above study. I have had the opportunity to consider the information, ask questions and have had these answered satisfactorily. ☐

2. I understand that my participation is voluntary and that I am free to withdraw at any time without giving any reason, without my medical care or legal rights being affected. ☐

3. I understand that relevant sections of my medical notes and data collected during the study, may be looked at by individuals from the University of Bath and the University of Bristol, from regulatory authorities or from the NHS Trust, where it is relevant to my taking part in this research. I give permission for these individuals to have access to my records. ☐

4. I agree to my General Practitioner being informed of my participation in the study. / I agree to my General Practitioner being involved in the study, including any necessary exchange of information about me between my GP and the research team. ☐

5. I understand that the information held and maintained by [insert site name] and the ADEPT-2 study team at the University of Bristol may be used to help contact me or ☐ rovide information about the results of the study. **[please continue to page 2]**

When completed: 1 for participant; 1 for researcher site file; 1 to be kept in medical notes.

OPTIONAL:

6. I agree to the audio recording of treatment sessions that may be shared with researchers at the University of Bath to ensure that the treatment is being delivered as intended. ☐

7. I agree to have my contact details shared with a qualitative researcher so they can contact me to arrange a study interview about my experience of the study and the support I have received. ☐

8. I agree to have my study interview audio recorded to help with analysis and I understand that my responses will remain anonymous. ☐

9. I agree that direct quotes and parts of voice modified audio-recordings from my study interview may be used for training, teaching, research and publication purposes and I understand that any quotes/modified audio-recordings used will remain anonymous. ☐

10. I understand that the information collected about me will be used to support other research in the future, and may be shared anonymously with other researchers. ☐

11. I give my consent to receive a copy of the results at the end of the study ☐

12. I give my consent to be contacted about other research opportunities in the future ☐

---

13. I agree to take part in the above study. ☐

____________________	____________________	____________________
Name of Participant	Date	Signature
____________________	____________________	____________________
Name of Person seeking consent	Date	Signature

When completed: 1 for participant; 1 for researcher site file; 1 to be kept in medical notes.
