# Supplementary material for: Effectiveness and cost-effectiveness of guided self-help for depression for autistic adults: the Autism Depression Trial (ADEPT-2) – protocol for a multicentre, randomised controlled trial of a remotely delivered low-intensity intervention
Source: BMJ Open. 2024 Nov 19;14(11):e084729. doi: 10.1136/bmjopen-2024-084729 (PMC11580278; doi:10.1136/bmjopen-2024-084729)
Supplement: Supplementary file 5 [file bmjopen-2024-084729.reviewer_comments.pdf]

PEER REVIEW HISTORY

BMJ Open publishes all reviews undertaken for accepted manuscripts. Reviewers are asked to complete a checklist review form and are provided with free text boxes to elaborate on their assessment. These free text comments are reproduced below.

ARTICLE DETAILS

Title (Provisional)

The effectiveness and cost effectiveness of Guided Self-Help for depression for autistic adults: the Autism Depression Trial (ADEPT-2): protocol for a multicentre, randomised controlled trial of a remotely delivered low-intensity intervention.

Authors

Mckeon, Holly Emily; Cotton, Leonora; Aldridge, Rona; Cape, Alison; Clout, Madeleine; Cooper, Kate; Dagnan, Dave; Dawn, Ed; Frost, Jessica; Georgakopoulou, Aikaterini; Garfield, Kirsty; horwood, jeremy; Ingham, Barry; Jervis, Vicky; Kessler, David; Langdon, Peter; Metcalfe, Chris; Rai, Dheeraj; Realpe, Alba; Russell, Christine; Sheridan, Hannah; Slowinska, Karolina; Thorn, Joanna; Wen, Liping; Wiles, Nicola; Russell, Ailsa

---

VERSION 1 - REVIEW

**Reviewer**	**1**
**Name**	**Blampied, Neville**
**Affiliation**	**University of Canterbury, School of Psychology, Speech, and Hearing**
**Date**	**09-Apr-2024**
**COI**	**I have no competing interests to declare**

The completion of this RCT will clearly improve our understanding of the CBT treatment of depression in Autistic people. I especially commend the involvement of Autistic people in co-design of the study following the pilot, and the inclusion of both quantitative and qualitative information from the carers. The following points represent essentially requests/suggestions for clarification of the protocol:

1. Who will triage the inclusion/exclusion decision (described as 'local researchers')? Is it the site PI, the GSH Coach, or some other person? It seems to me that this decision will require a high level of clinical expertise (e.g., assessing suicide risk) based on both an interview (face-to-face) and the information supplied by the potential participant in the EOI. Will any steps be taken to check information in any EOI with carers if there is concern about the quality of the EOI information?

2. The GSH Intervention: Does this include any explicit homework assignment? Given that Behavioural Activation requires actual activation to work, homework, and the checking thereof, would seem to be important.

3. Statistical Analysis: I understand that a more complete description of the statistical analysis will be published in due course. I note that the analysis will be based on Intention to Treat. This raises the important question of how missing data will be handled, a complex and challenging question that will need specification and justification. In addition to ITI analyses I would recommend an analysis of protocol completers (those who received the minimal dose), since what we actually want to know is does the treatment work for those who actually received the treatment.

4. I would also recommend the inclusion of a consistency analysis. Consistency analyses are rarely performed in clinical psychology RCTs but they address the important question of the extent to which improvement on the primary outcome (depression in this case) is associated with improvements in other aspects of the participants condition and wellbeing. This information does not emerge from statistical analyses performed DV by DV. There are several ways of conducting such analyses, a simple one being to correlate gain scores on the primary outcome DV with gain scores on the secondary DVs. A moderate or greater positive correlation between, e.g., improvement in depression and improvement in anxiety, suggests that the therapy has has a moderately consistent effect on at least some aspects of wellbeing (e.g., anxiety).

5. I would also recommend that you consider using the Reliable Change Index to classify each participant at each outcome assessment point as reliably changed, reliably deteriorated, or indeterminate. This takes into account the precision of measurement of the DVs. The %Reliably Changed is a useful effect size measure.

6. I assume that the data analysis will also include some investigation of moderators of treatment outcome? Again, this is important clinical information.

Some minor points in the manuscript:

1. Setting: Manuscript says "6" but only 5 are listed.

2. TAU. Participants are 'signposted' to treatment, but do they actually receive treatment? Will the different forms of TAU received be recorded (including no treatment) and be analysed?

3. Blinding. Is there a provision for unblinding if some issue of clinical concern arises (e.g., threat of suicide). The ms is unclear about when unblinding might occur and who would make the decision.

4. P14 l 19. The sentence beginning '/the ...' needs editing for punctuation and grammar.

Reviewer 2

Name **Williams, Zachary**

Affiliation **Vanderbilt University School of Medicine, Medical Scientist Training Program**

Date **30-Apr-2024**

COI **I have received consulting fees from Roche related to the conduct of clinical trials for autism.**

I thank the editor for giving me the chance to review this protocol, and I sincerely apologize for taking so long to do so. The authors present an overview and study protocol for ADEPT-2, an effectiveness and cost-effectiveness RCT in which a guided self-help intervention is tested as a low-intensity yet scalable option that may potentially improve depressive symptoms in autistic adults. The protocol is extremely well-described, and I generally have very few modifications to suggest. My feedback is relatively minor, and you can find these comments below. I very much look forward to reading the full study when it is published!

1. In citing other support for CBT to treat depression, there is now a reasonably large DBT trial that was published in the last month (while this sitting waiting to be reviewed by me—sorry!), demonstrating durable effects of (full-model) DBT on depressive symptoms in suicidal autistic adults (compared to a fairly rigorous TAU standard of weekly-or-more eclectic suicidality-focused psychotherapy): https://www.cambridge.org/core/journals/psychological-medicine/article/effectiveness-and-safety-of-dialectical-behavior-therapy-for-suicidal-ideation-and-behavior-in-autistic-adults-a-pragmatic-randomized-controlled-trial/4464CA3C0D3DDEF5F3A4BD45415B9B50

- It would be worthwhile to add this paper to the list of citations for studies of CBT for depression in autism. Obviously, full-model DBT is a little more than "just" CBT, but it's relevant to include.

- My group has also published a small single-group trial of an adapted group CBT intervention for depression in autistic adolescents, if that's evidence enough (i.e., without a control intervention) to demonstrate some relevance of adapted CBT interventions to the treatment of depression in autism. Up to you as to whether you'd like to include it (no pressure): https://doi.org/10.1177/13623613231213543

2. If the authors are interested, it may even be useful to consider calculating the autism-specific BDI-II latent trait scores (based on IRT model parameters in the prior psychometric study published by my group: see https://asdmeasures.shinyapps.io/bdi_score/). The assessment of these scores and their SE's over time allows one to calculate the IRT-based reliable change index, i.e., (Z2 - Z1) /sqrt(SE.Z2^2 + SE.Z1^2), which is in Z-score metric that can be used to determine whether an individual has made significant pre-post change not due to unreliability of the measure. (Example of this metric in use: https://doi.org/10.1080/10503307.2013.794400).

- This wouldn't necessarily have to be a primary analysis or anything, but to determine the subset of participants who achieved "reliable" change on the BDI-II is similar to a "responder" analysis in many other trials, using an empirically derived cut-point for the designation of how much change to consider meaningful (i.e., an amount that's based on how precise the measure is).

3. I very much appreciate the use of reimbursement for completion of follow-up measures as a way to reduce the impact of non-random missingness on the data. However, I don't believe that the reimbursement amounts are ever described, which would be helpful for readers to fully understand the protocol (and if measure completion was low despite this level of reimbursement, perhaps the size of the payments could be scrutinized).

4. The six sites under "setting" are listed as five points because the "North of England" line doesn't seem to have a carriage return before it. This seems to just be a typo, but if it is not, I'd want to know why there are only five regions listed.

5. The trial specifically does not appear to note in its inclusion or exclusion criteria the cognitive ability of the autistic people taking part (just their English/Welsh literacy). I presume this means that the trial was indeed open to autistic individuals with intellectual disabilities, provided they were able to read and understand the treatment materials well enough as judged by the study team. If this was the case, I think it's actually worthwhile to ensure that the reader is aware that the study was designed to extend down into the intellectual disability population (i.e., in inclusion criterion one can note "with or without intellectual disability" alongside the "clinical diagnosis of autism spectrum disorder").

- Presumably genetic conditions underlying one's autism would also not disqualify an individual (e.g., Fragile X, Tuberous Sclerosis, 22q deletion), provided epilepsy was not an active comorbidity—this may also be relevant to state outright.

6. I would appreciate a slight bit more detail on the composition of he advisory group (e.g., how many autistic adults vs. carers vs. clinicians (only that one autistic CBT therapist?). Also, if there is a set (i.e., structured) amount of engagement (e.g., quarterly meetings) with the advisory group, I would note that in the protocol under the patient/public involvement section as well, as that is part of your protocol.

Overall, I find this protocol in excellent shape, and I hope to see it published soon.

- Zack Williams

VERSION 1 - AUTHOR RESPONSE

Reviewer: 1
Prof. Neville Blampied, University of Canterbury
Comments to the Author:
The completion of this RCT will clearly improve our understanding of the CBT treatment of depression in Autistic people. I especially commend the involvement of Autistic people in co-

design of the study following the pilot, and the inclusion of both quantitative and qualitative information from the carers. The following points represent essentially requests/suggestions for clarification of the protocol:

1. Who will triage the inclusion/exclusion decision (described as 'local researchers')? Is it the site PI, the GSH Coach, or some other person? It seems to me that this decision will require a high level of clinical expertise (e.g., assessing suicide risk) based on both an interview (face-to-face) and the information supplied by the potential participant in the EOI. Will any steps be taken to check information in any EOI with carers if there is concern about the quality of the EOI information?

We thank the reviewer for their comments.

Screening for eligibility criteria is first reviewed by the central team. Those potentially eligible are passed onto site, where the local research assistant will complete screening, and confirm all inclusion/exclusion criteria in the baseline appointment. Standardised measures are used such as the PHQ-9 to screen for suicide risk. The site PI then reviews this information and makes a final decision regarding eligibility. Potentially eligible individuals known to healthcare services at site will be asked to give permission for professionals involved in their care to be contacted for further consideration of suitability of taking part in the study in the context of broader service delivery.

We have added to the manuscript as follows:

"Potentially eligible individuals will be invited to attend an appointment with a suitable participating site during which the local researcher will answer any questions, confirm eligibility **criteria**, receive written informed econsent (if the individual decides to take part) and complete any outstanding baseline data collection. **Potentially eligible individuals known to healthcare services at site i.e. with a current electronic healthcare record, will be asked to give permission for professionals involved in their care to be contacted for further consideration of suitability of taking part in the study in the context of broader service delivery. The PI reviews all information gathered at baseline and makes the final decision regarding participant eligibility and randomisation."**

Participants who endorse a score of 3 on item 9 of the PHQ-9 will be followed-up by the site lead clinical researcher (PI) to assess suicide risk.

We have added to the manuscript:

"Risk of suicide or severity of depression such that a low intensity psychological intervention is not clinically indicated, **as judged by the site lead clinical researcher. Participants who endorse a score of 3 on Item 9 of the PHQ-9 will be followed-up to assess suicide risk.**"

2. The GSH Intervention: Does this include any explicit homework assignment? Given that Behavioural Activation requires actual activation to work, homework, and the checking thereof, would seem to be important.

Between session tasks are outlined in the intervention materials for each session and a personalised plan developed with GSH coach support at each session. Engagement with and

completion of between sessions tasks is reviewed at each session and recorded in the GSH coach record form.

We have added to the manuscript:

Between session tasks are suggested to consolidate the treatment principles, **“and this is checked for completion and quality by the coach at the next appointment.”**

3. Statistical Analysis: I understand that a more complete description of the statistical analysis will be published in due course. I note that the analysis will be based on Intention to Treat. This raises the important question of how missing data will be handled, a complex and challenging question that will need specification and justification. In addition to ITI analyses I would recommend an analysis of protocol completers (those who received the minimal dose), since what we actually want to know is does the treatment work for those who actually received the treatment.

We agree with the reviewer that there will be interest in whether participants who attend more sessions experience greater benefit, although the per protocol analysis suggested will be subject to strong biases - e.g. reverse causality where it is the participants who experience improvements in their depression who are more likely to continue complete all the sessions offered. We have added to the manuscript:

"To investigate the correlation between attending sessions and the primary outcome response, we will present summary statistics for the 16-week BDI-II for the intervention group participants who attend 0, 1-5 and 6 or more sessions. A sensitivity analysis will repeat the primary analysis on a complier average causal effect basis, comparing intervention group participants attending one or more sessions against the estimated outcome of the comparable participants allocated to the comparison group (i.e. those comparison group participants who would have attended one or more sessions had they instead been allocated to the intervention)."

4. I would also recommend the inclusion of a consistency analysis. Consistency analyses are rarely performed in clinical psychology RCTs but they address the important question of the extent to which improvement on the primary outcome (depression in this case) is associated with improvements in other aspects of the participants condition and wellbeing. This information does not emerge from statistical analyses performed DV by DV. There are several ways of conducting such analyses, a simple one being to correlate gain scores on the primary outcome DV with gain scores on the secondary DVs. A moderate or greater positive correlation between, e.g., improvement in depression and improvement in anxiety, suggests that the therapy has has a moderately consistent effect on at least some aspects of wellbeing (e.g., anxiety).

We will present the estimated intervention effects on the primary and secondary measures of clinical outcome. It will hence be clear if the benefits of the intervention extend beyond depression, to co-morbid anxiety for example. We prefer not to present correlations as suggested as the interpretation will be impeded due to not all participants having (for example) co-morbid anxiety, a common response set across the questionnaire measures, and the action of any non-specific factors such as spontaneous recovery which a correlation analysis will not control.

5. I would also recommend that you consider using the Reliable Change Index to classify each participant at each outcome assessment point as reliably changed, reliably deteriorated, or indeterminate. This takes into account the precision of measurement of the DVs. The %Reliably Changed is a useful effect size measure.

Thank you for this suggestion. We will illustrate individual participant changes on the primary outcome variable by plotting the 16-week assessment of the BDI-II against the baseline assessment, for each of the allocated groups on separate scatterplots. We will highlight individual changes that exceed the minimum clinically important difference.

6. I assume that the data analysis will also include some investigation of moderators of treatment outcome? Again, this is important clinical information.

This is correct, subgroup analyses are included as below and will be listed in the Statistical Analysis Plan in detail. This will be added to the ISRCTN registry once finalised.

The baseline characteristics investigated for subgroup analyses are:

- CIS-R Anxiety disorder as primary diagnosis
- Baseline depression severity
- Structured occupation (defined as any employment or student vs. unemployed)
- Therapists who deliver the intervention with greater than two participants

Some minor points in the manuscript:
1. Setting: Manuscript says "6" but only 5 are listed.

Thank you for noting this. The second site was listed on the bullet point of the first and has now been reformatted.

2. TAU. Participants are 'signposted' to treatment, but do they actually receive treatment? Will the different forms of TAU received be recorded (including no treatment) and be analysed?

Participants allocated to TAU will have the opportunity to report this in their follow up questionnaires at 16-, 32- and 52- weeks post randomisation. As part of the health economics analysis, all participants will be asked how many times they have had NHS counselling or any other “talking therapy” at a GP surgery or health centre, or alternatively over the telephone or online, within the last 4 months (excluding GSH received in the study). These data will not be included in a statistical model for the main trial dataset.

3. Blinding. Is there a provision for unblinding if some issue of clinical concern arises (e.g., threat of suicide). The ms is unclear about when unblinding might occur and who would make the decision.

Thank you for this.

We have added to the manuscript:

"Participant unblinding is only required if information about allocation would affect the clinical response to crisis such as risk of suicide. Unblinding will be carried out by the central trial management team or local RAs in such situations."

4. P14 l 19. The sentence beginning '/the ...' needs editing for punctuation and grammar.

Thank you for this, this sentence has been amended in the manuscript as follows:

"~~/~~The Data Monitoring Committee (DMC) ~~meet~~ are an independent committee who assess the safety and efficacy of the trial's interventions, ~~and to~~ monitor the trial's overall conduct, and protect its validity and credibility."

Reviewer: 2
Mr. Zachary Williams, Vanderbilt University School of Medicine, Vanderbilt University Medical Center
Comments to the Author:
I thank the editor for giving me the chance to review this protocol, and I sincerely apologize for taking so long to do so. The authors present an overview and study protocol for ADEPT-2, an effectiveness and cost-effectiveness RCT in which a guided self-help intervention is tested as a low-intensity yet scalable option that may potentially improve depressive symptoms in autistic adults. The protocol is extremely well-described, and I generally have very few modifications to suggest. My feedback is relatively minor, and you can find these comments below. I very much look forward to reading the full study when it is published!

1. In citing other support for CBT to treat depression, there is now a reasonably large DBT trial that was published in the last month (while this sitting waiting to be reviewed by me—sorry!), demonstrating durable effects of (full-model) DBT on depressive symptoms in suicidal autistic adults (compared to a fairly rigorous TAU standard of weekly-or-more eclectic suicidality-focused psychotherapy): https://www.cambridge.org/core/journals/psychological-medicine/article/effectiveness-and-safety-of-dialectical-behavior-therapy-for-suicidal-ideation-and-behavior-in-autistic-adults-a-pragmatic-randomized-controlled-trial/4464CA3C0D3DDEF5F3A4BD45415B9B50
- It would be worthwhile to add this paper to the list of citations for studies of CBT for depression in autism. Obviously, full-model DBT is a little more than "just" CBT, but it's relevant to include.
- My group has also published a small single-group trial of an adapted group CBT intervention for depression in autistic adolescents, if that's evidence enough (i.e., without a control intervention) to demonstrate some relevance of adapted CBT interventions to the treatment of depression in autism. Up to you as to whether you'd like to include it (no pressure):
https://doi.org/10.1177/13623613231213543

We thank the reviewer for these suggestions. Both have been added to the manuscript.

2. If the authors are interested, it may even be useful to consider calculating the autism-specific BDI-II latent trait scores (based on IRT model parameters in the prior psychometric study published by my group: see https://asdmeasures.shinyapps.io/bdi_score/). The assessment of these scores and their SE's over time allows one to calculate the IRT-based reliable change index, i.e., (Z2 - Z1) /sqrt(SE.Z2^2 + SE.Z1^2), which is in Z-score metric that can be used to determine whether an individual has made significant pre-post change not due to unreliability of

the measure. (Example of this metric in use: https://doi.org/10.1080/10503307.2013.794400).
- This wouldn't necessarily have to be a primary analysis or anything, but to determine the subset of participants who achieved "reliable" change on the BDI-II is similar to a "responder" analysis in many other trials, using an empirically derived cut-point for the designation of how much change to consider meaningful (i.e., an amount that's based on how precise the measure is).

We agree with both reviewers that there will be interest in the responses of individual participants, and now plan the presentation described in our response to reviewer 1's major comment #5. We strongly prefer to interpret individual changes in comparison with the minimum clinically important difference, as we believe this will be better understood by clinicians and participants, compared to a cut-off for the "statistical significance" of individual changes.

3. I very much appreciate the use of reimbursement for completion of follow-up measures as a way to reduce the impact of non-random missingness on the data. However, I don't believe that the reimbursement amounts are ever described, which would be helpful for readers to fully understand the protocol (and if measure completion was low despite this level of reimbursement, perhaps the size of the payments could be scrutinized).

We thank the reviewer for this suggestion, and have added to the manuscript:

"Participants are offered a £10.00 gift voucher to thank them for their time after the completion of each of the 4 questionnaires."

4. The six sites under "setting" are listed as five points because the "North of England" line doesn't seem to have a carriage return before it. This seems to just be a typo, but if it is not, I'd want to know why there are only five regions listed.

Thank you for noting this. The second site was listed (North of England) on the bullet point of the first and has now been reformatted.

5. The trial specifically does not appear to note in its inclusion or exclusion criteria the cognitive ability of the autistic people taking part (just their English/Welsh literacy). I presume this means that the trial was indeed open to autistic individuals with intellectual disabilities, provided they were able to read and understand the treatment materials well enough as judged by the study team. If this was the case, I think it's actually worthwhile to ensure that the reader is aware that the study was designed to extend down into the intellectual disability population (i.e., in inclusion criterion one can note "with or without intellectual disability" alongside the "clinical diagnosis of autism spectrum disorder").
- Presumably genetic conditions underlying one's autism would also not disqualify an individual (e.g., Fragile X, Tuberous Sclerosis, 22q deletion), provided epilepsy was not an active comorbidity—this may also be relevant to state outright.

Thank you for noting this. This item in the full exclusion in the main protocol elaborates as follows: "This will be established by reviewing the case notes for record of cognitive/educational assessment indicating significant literacy difficulties, on information provided by the referring clinician and/or during the eligibility assessment when it is difficult to gain consent to participate in the research because of difficulties reading and thus comprehending the study

information sheet. We will strive to include all adults in the study if supporters are available to help an individual access the treatment where written/spoken English, non-English & Welsh presents a barrier."

This section in the manuscript has been updated to clarify, the below has been added:

"We will strive to include all adults in the study if supporters are available to help an individual access the treatment where written/spoken English, non-English & Welsh presents a barrier."

6. I would appreciate a slight bit more detail on the composition of the advisory group (e.g., how many autistic adults vs. carers vs. clinicians (only that one autistic CBT therapist?). Also, if there is a set (i.e., structured) amount of engagement (e.g., quarterly meetings) with the advisory group, I would note that in the protocol under the patient/public involvement section as well, as that is part of your protocol.

Thank you for this comment. The advisory group has involved different contributors throughout the trial and therefore we have not described this in detail here. To date, we have had the input of one autistic CBT therapist, one carer and seven autistic adults.
Similarly, engagement of the group has varied according to study stage with more frequent meetings during the study set-up and final completion stages. The advisory group have also worked in smaller groups at times according to expertise and interest e.g. a pair of experts by experience meeting independently to prepare study newsletters, We estimate that advisory group meetings occur on average every 2 months across the duration of the study.

Overall, I find this protocol in excellent shape, and I hope to see it published soon.

- Zack Williams

Reviewer: 1
Competing interests of Reviewer: I have no competing interests to declare

Reviewer: 2
Competing interests of Reviewer: I have received consulting fees from Roche related to the conduct of clinical trials for autism.
